# Effect of β3‐adrenergic receptor gene polymorphism and lifestyle on overweight Japanese rural residents: A cross‐sectional study

**DOI:** 10.1002/osp4.560

**Published:** 2021-09-21

**Authors:** Akinori Hara, Phat Minh Nguyen, Hiromasa Tsujiguchi, Masaharu Nakamura, Yohei Yamada, Keita Suzuki, Fumihiko Suzuki, Tomoko Kasahara, Oanh Kim Pham, Haruki Nakamura, Yasuhiro Kambayashi, Yukari Shimizu, Thao Thi Thu Nguyen, Sakae Miyagi, Takayuki Kannon, Takehiro Sato, Kazuyoshi Hosomichi, Atsushi Tajima, Hiroyuki Nakamura

**Affiliations:** ^1^ Department of Hygiene and Public Health Faculty of Medicine Institute of Medical, Pharmaceutical and Health Sciences Kanazawa University Kanazawa Ishikawa Japan; ^2^ Community Medicine Support Dentistry Ohu University Hospital Koriyama Fukushima Japan; ^3^ Department of Public Health Faculty of Veterinary Medicine Okayama University of Science Imabari Ehime Japan; ^4^ Department of Nursing Faculty of Health Sciences Komatsu University Komatsu Ishikawa Japan; ^5^ Department of Epidemiology Faculty of Public Health Haiphong University of Medicine and Pharmacy Hai Phong Vietnam; ^6^ Division of Biostatistics Innovative Clinical Research Center Kanazawa University Kanazawa Ishikawa Japan; ^7^ Department of Bioinformatics and Genomics Faculty of Medicine Institute of Medical, Pharmaceutical and Health Sciences Kanazawa University Kanazawa Ishikawa Japan

**Keywords:** BDHQ, obesity, single nucleotide polymorphism, *β*3‐adrenergic receptor

## Abstract

**Objectives:**

The β3‐adrenergic receptor (*ADRB3*) gene polymorphism has been implicated in obesity. Therefore, the contribution of *ADRB3* Trp64Arg polymorphism to obesity‐related indicators was investigated, taking into account the lifestyle‐related factors in a Japanese rural population.

**Methods:**

A total of 600 Japanese adults aged ≥40 years in a population‐based cohort study were analyzed. The *ADRB3* polymorphism was determined using peripheral blood samples. Associations between genotype and body mass index (BMI), waist circumference (WC), and body fat (BF) percentage were examined, adjusting for lifestyle‐related factors, including daily nutrient intake.

**Results:**

The frequency of Arg64 allele carriers was 36%. There was no significant difference in BMI, WC, or BF between the groups with or without the Trp64Arg polymorphism. Multivariable logistic regression analysis showed that the Trp64Arg polymorphism was not associated with these three indicators, but lifestyle factors including physical inactivity, higher energy and sodium consumption, and less animal protein intake were significantly related to increased WC and BF percentages.

**Conclusions:**

The Trp64Arg polymorphism of *ADRB3* gene did not contribute to increased BMI, WC, or BF. However, lifestyle‐related factors impacted these indicators in middle‐aged and older Japanese individuals living in rural areas.

## 1 INTRODUCTION

Long‐term energy imbalance, that is, an abundance of food, low physical activity, and several other environmental factors that interact with the genetic susceptibility of an individual to produce a positive energy balance, is recognized as a fundamental contributor to excess body weight.^1^, ^2^ In addition to overeating and inactivity, increasing evidence suggests that the quality of dietary fats and carbohydrates is important for maintaining weight.^3^ Moreover, other environmental factors, biological factors (including lack or low quality of sleep and psychological factors), and sociocultural factors (including older age of first‐time mothers as well as social stress) could also affect obesity.^2^

Among obesity‐related genetic factors in humans, the β3‐adrenergic receptor (ADRB3), which is mainly expressed in adipose tissue, plays an essential role in energy metabolism through thermogenesis and regulation of lipolysis in the adipose tissue.^4^ Walston et al.^5^ first reported a genetic variant of ADRB3 that carries a tryptophan‐to‐arginine missense mutation at the amino acid position 64 (Trp64Arg), resulting in a lower tendency to achieve a resting metabolic rate. Following this initial report, multiple studies have described an association of the Trp64Arg variant with overweight or obesity^6^, ^7^, ^8^, ^9^, ^10^, ^11^ and related comorbidities, such as metabolic syndrome,^12^ type 2 diabetes,^13^ and hypertension,^14^ although the results were conflicting.

In Japan, a previous study reported that while the frequency of the Trp64Arg allele was similar in participants with and without obesity (23% vs. 22%), reduction in body weight in mutation‐carrier participants with obesity was lower than that in non‐carrier participants after three months of treatment with a combined low‐calorie diet and exercise regimen.^15^ Therefore, it has been suggested that the Trp64Arg variant of *ADRB3* might predict the problems associated with weight loss by women with obesity.^15^ Another study reported that the frequency of Trp64Arg in participants with obesity was significantly higher than that in participants without obesity. Moreover, the presence of this mutation was accompanied by higher levels of fasting and 2‐h serum insulin in a glucose tolerance test.^16^

However, other studies have reported that the Trp64Arg mutation of ADRB3 is not associated with obesity or its comorbidities, even in the same races. For example, a longitudinal study did not demonstrate significant differences in weight changes between the genotype groups in 746 individuals from the general Japanese population.^17^ Another study also did not report a significant relationship between the Trp64Arg polymorphism and the frequency of metabolic syndrome among 1416 individuals from the general Japanese population.^12^

As mentioned in a meta‐analysis report, in addition to the number and ethnicity of the study participants, differences in comorbidities and related environmental factors (such as dietary habits and physical activities) in regions where each study was conducted could account for these inconsistent findings.^11^ Furthermore, a limited number of epidemiological studies are available on unselected populations, which examined genetic and environmental factors that could affect body weight and fat mass.

Here, the prevalence of ADRB3 Trp64Arg polymorphism and the effect of this polymorphism on obesity‐related indicators are described, taking into account major lifestyle‐related factors in a Japanese rural population.

## 2 MATERIALS AND METHODS

### 2.1 Study design and participants

This cross‐sectional study was a part of the Shika study, a longitudinal observational community‐based study involving residents of the town of Shika, as described in details elsewe.^18^, ^19^ Briefly, 19,348 individuals (9225 men and 10,123 women with a mean age of 55.2 years) in June 2021, of which approximately 44% and 74% were at least 65 and 40 years old, respectively, were included in the study. A self‐administered questionnaire was used, and health examination data were collected between 2013 and 2017.

**FIGURE 1:**
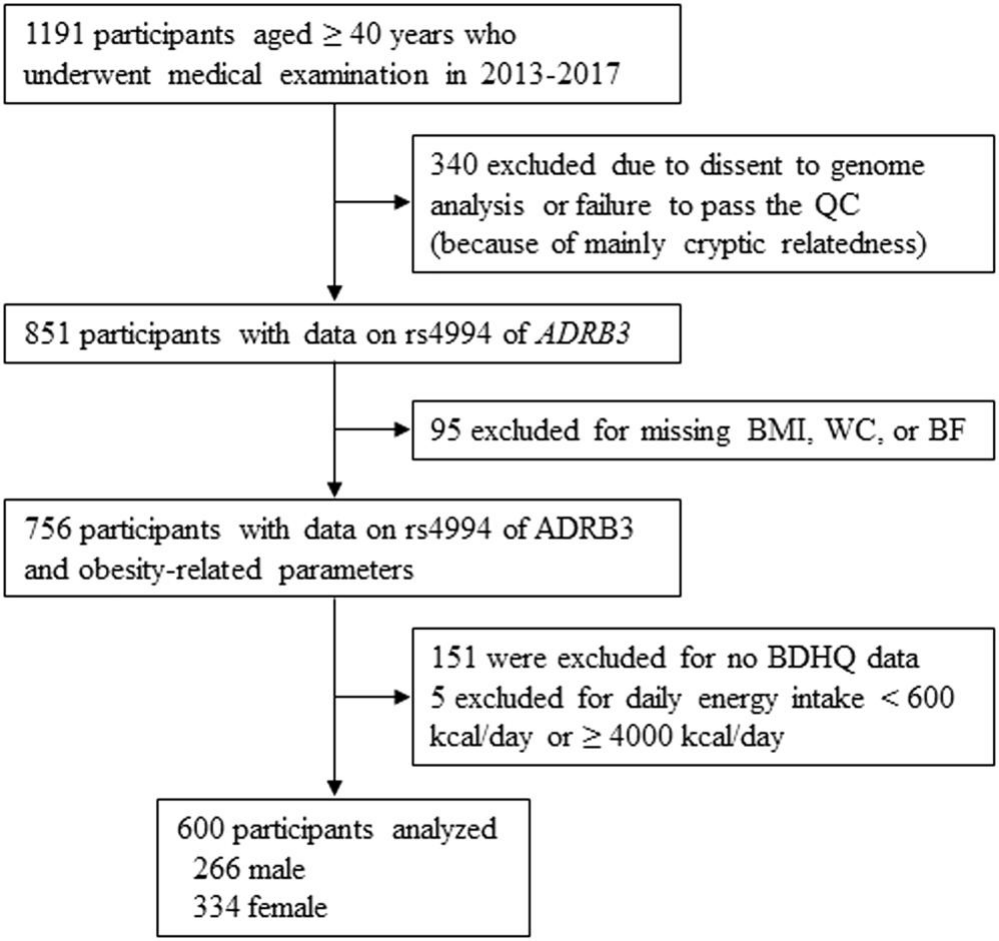
Flow diagram showing the study enrollment procedure. *ADRB3*, β3‐adrenergic receptor; BDHQ, brief self‐administered diet history questionnaire; BF, body fat; BMI, body mass index; QC, quality control; WC, waist circumference

The study enrollment procedure is outlined in Figure 1. Of the 1191 participants, aged ≥40 years, who underwent a medical examination during the study period, 586 participants were excluded due to the lack of data related to ADRB3 (n = 340), obesity indices (n = 95), or nutrient status (n = 151). After excluding five subjects with a daily energy intake of <600 or >4000 kcal/day,^20^ data obtained from 600 individuals were analyzed. The Shika study was approved by the Medical Ethics Committee of Kanazawa University (approval number: 1491). Informed consent was obtained from all the participants.

### 2.2 Obesity indices

Body mass index (BMI) and body fat (BF) percentage were measured using a body composition analyzer (Tanita BC‐118D scale). Waist circumference (WC) of the participants was measured at the umbilical level with a tape measure. Triplicate measurements were obtained and the average value was calculated. Participants were divided into two subgroups taking into account their BMI values according to the classification of the Japan Society for the Study of Obesity: non‐overweight/obese (BMI < 25 kg/m^2^) and overweight/obese (BMI ≥25 kg/m^2^).^21^ In addition, the participants were divided into two subgroups according to WC: non‐overweight (men, <85 cm; women, <90 cm) and overweight (men, ≥85 cm; women, ≥90 cm).^21^ Furthermore, the participants were divided into two subgroups according to the BF percentage: non‐overweight (men, <25%; women, <35%) and overweight (men, ≥25%; women, ≥35%).^22^

### 2.3 Genotyping

Genomic DNA was extracted from blood samples using the services of SRL, Inc. (Tokyo, Japan) or using the QIAamp DNA Blood Maxi Kit (Qiagen, Hilden, Germany). Genome‐wide single nucleotide polymorphism (SNP) genotyping was performed in 1150 individuals who consented to genome analysis, using the Japonica Array v2^23^ (Toshiba Co. Ltd., Tokyo, Japan). Quality control (QC) procedures based on factors, such as gender identity between karyotype and questionnaire, SNP call rates, Hardy‐Weinberg equilibrium test, inbreeding coefficient, cryptic relatedness, and population structure were applied to the array data. Data on ADRB3 Trp64Arg (rs4994) genotypes obtained from 851 unrelated individuals (based on genome‐wide$\;\hat{\pi }$ values) that passed the QC test were extracted from the array data. In the QC procedure, the SNP call rate was 100%, and a departure from the Hardy‐Weinberg equilibrium was not observed.

### 2.4 Nutritional assessment

A brief self‐administered dietary history questionnaire (BDHQ) was used to estimate nutrient intake by the participants. The BDHQ was developed based on a comprehensive Japanese version of a standard food frequency questionnaire.^24^ The BDHQ validity, as confirmed by other studies, was considered to exhibit a satisfactory ranking ability for several nutrients in the Japanese population.^25^, ^27^ Moreover, the estimated protein and fat intake from animal and vegetable sources were used separately in the present analysis. To obtain improved values, which display better correlation with dietary records as compared to the use of crude values, the density method (% energy or per 1000 kcal of daily energy intake) was employed to measure the daily consumption of each nutrient. In a previous study, correlation coefficients between the dietary record‐based amounts and BDHQ‐estimated energy‐adjusted intakes of nutrients were as follows: 0.49 for proteins, 0.65 for fats, 0.67 for carbohydrates, 0.61 for sodium, 0.67 for calcium, and 0.76 for fiber in women, and 0.62, 0.70, 0.79, 0.60, 0.77, and 0.79 for proteins, fats, carbohydrates, sodium, calcium, and fiber, respectively, in men.^25^

### 2.5 Other variables

Other variables, such as age, sex, smoking status, frequency of exercise, and alcohol consumption were assessed using self‐administered questionnaires. Smoking status was classified as non‐smoker, ex‐smoker, or current smoker.^27^ Habitual alcohol consumption was defined as drinking more than one glass of sake (containing 22 g ethanol) per day at least three times a week.^27^ The frequency of exercise was estimated as follows: the participants were asked whether they had exercised for more than 30 min at least twice a week during the previous year or had performed tasks such as walking, cleaning, and carrying baggage for more than 1 h per day.^27^ Participants with affirmative responses to either of these questions were considered to have exercised at an adequate level based on the World Health Organization guidelines on physical activity.^28^

### 2.6 Statistical analysis

Descriptive characteristics at baseline were compared according to each obesity index. The means of continuous variables were compared using the Student’s *t*‐test. Deviation of genotype distributions from the Hardy‐Weinberg equilibrium and comparison of the proportions of categorical variables were examined using the Chi‐square test. Analysis of variance was used to assess the differences in anthropometric characteristics among *ADRB3* gene polymorphisms. Multivariable logistic regression was applied to assess the association between the Trp64Arg variant and each obesity index, with adjustment for other known variables. These variables, which were used as covariates, included age, gender, and lifestyle‐related factors (smoking status, drinking habits, and exercise). Moreover, dietary variables including total energy consumption and individual consumption of proteins, lipids, carbohydrates,^3^ sodium,^29^ calcium,^30^ and fiber,^31^, ^32^ as well as lifestyle‐related factors were also selected based on previous studies. Furthermore, to reduce noise variables, a stepwise selection with backward elimination of predictors from the above‐mentioned model was applied. The SPSS version 23 (IBM Corp., Tokyo, Japan) was used for all analyses. Differences were considered statistically significant at *p* < 0.05.

## 3 RESULTS

### 3.1 Characteristics of participants

The characteristics of the study population are summarized in Table 1. The mean age of the study participants was 62.0 years (SD ± 10.9). A total of 334 women were included in the study, accounting for 56% of the total participants. The proportion of participants with habitual alcohol consumption and the percentage of participants exercising at an adequate level were 23% and 58%, respectively. The percentages of current smokers and former smokers were 20% and 26%, respectively. A significant difference was observed in the smoking status of former smokers between overweight/obese and non‐overweight/obese groups based on BMI (32% vs. 23%, respectively, *p* = 0.009) and WC (41% vs. 18%, respectively, *p* < 0.001). Significant differences in BMI were observed between males and females (54% vs. 41%, respectively, *p* = 0.003), as well as in daily total energy intake (1950 kcal/day vs. 1823 kcal/day, *p* = 0.025) and dietary fiber (6.2 g/1000 kcal vs. 6.7 g/1000 kcal, *p* = 0.012) between overweight/obese and non‐overweight/obese groups. Regarding WC, significant differences in sex distribution (male: 72% vs. females: 30%, *p* < 0.001), as well as in drinking habits (31% vs. 19%, *p* = 0.001) and intake of proteins (14.6%E vs. 15.6%E, *p* < 0.001), fats (23.8%E vs. 25.4%E, *p* = 0.003), and calcium (270 mg/1000 kcal vs. 305 mg/1000 kcal, *p* < 0.001), as well as total energy (2041 kcal/day vs. 1765 kcal/day, *p* < 0.001) and dietary fiber (6.1 g/1000 kcal vs. 6.8 g/1000 kcal, *p* < 0.001) were observed between overweight and non‐overweight groups, respectively. Furthermore, significant differences were detected between age groups (64 years vs. 60 years, *p* < 0.001) as well as in drinking habits (19% vs. 27%, *p* = 0.023) between overweight and non‐overweight groups, respectively, according to the BF percentage.

**TABLE 1:** Participant characteristics with or without overweight

	BMI					Waist circumference					Body fat				
	Non‐overweight/obese (*n* = 436)		Overweight/obese (*n* = 164)		*p*‐value	Non‐overweight (*n* = 399)		Overweight (*n* = 201)		*p*‐value	Non‐overweight (*n* = 330)		Overweight (*n* = 270)		*p*‐value
	Mean/*N*	SD/%	Mean/*N*	SD/%		Mean/*N*	SD/%	Mean/*N*	SD/%		Mean/*N*	SD/%	Mean/*N*	SD/%	
Age (years)	62.03	10.70	62.02	11.60	0.99	62.04	10.98	62	10.91	0.97	60.44	10.5	63.96	11.18	**<0.001**
Male, *N* (%)	177	40.6	89	54.3	**0.003**	121	30.3	145	72.1	**<0.001**	149	45.2	117	43.3	0.66
Smoking status					**0.009**					**<0.001**					0.11
Never, *N* (%)	252	57.8	72	43.9	‐	258	64.7	66	32.8	‐	176	53.3	148	54.8	‐
Former, *N* (%)	102	23.4	53	32.3	‐	72	18.0	83	41.3	‐	78	23.6	77	28.5	‐
Current, *N* (%)	82	18.8	39	23.8	‐	69	17.3	52	25.9	‐	76	23	45	16.7	‐
Regular exercise, *N* (%)	257	58.9	88	53.7	0.24	243	60.9	102	50.7	**0.018**	190	57.6	155	57.4	0.97
Drinking habits, *N* (%)	98	22.5	41	25	0.52	76	19.0	63	31.3	**0.001**	88	26.7	51	18.9	**0.023**
Dietary intake															
Total energy (kcal/day)	1823.44	599.00	1949.56	645.42	**0.025**	1765.46	583.92	2041.43	632.40	**<0.001**	1858.76	593.02	1856.88	639.97	0.97
Total protein (%E)	15.38	3.23	14.92	3.22	0.12	15.58	3.30	14.63	3.01	**<0.001**	15.22	3.23	15.31	3.24	0.75
Animal protein (%E)	8.91	3.23	8.59	3.35	0.28	9.05	3.31	8.36	3.13	**0.014**	8.80	3.22	8.85	3.32	0.83
Vegetable protein (%E)	6.48	1.10	6.33	1.05	0.16	6.52	1.11	6.27	1.04	**0.007**	6.42	1.05	6.45	1.13	0.75
Total fat (%E)	24.99	5.90	24.61	6.46	0.50	25.41	6.09	23.84	5.86	**0.003**	24.59	5.98	25.24	6.14	0.19
Animal fat (%E)	11.88	3.92	11.70	4.17	0.63	12.13	4.02	11.23	3.86	**0.009**	11.73	3.95	11.96	4.04	0.48
Vegetable fat (%E)	13.11	3.59	12.91	4.08	0.56	13.28	3.75	12.61	3.66	**0.037**	12.86	3.61	13.29	3.87	0.17
Carbohydrate (%E)	53.79	8.43	53.54	8.74	0.75	53.92	8.37	53.35	8.78	0.44	53.85	8.41	53.57	8.64	0.69
Sodium (mg/100 kcal)	2457.43	543.24	2432.76	532.69	0.62	2460.62	543.17	2430.95	534.58	0.53	2438.45	540.51	2465.64	540.11	0.54
Calcium (mg/1000 kcal)	297.19	111.46	282.28	100.82	0.14	304.85	113.69	269.82	94.34	**<0.001**	285.55	107.75	302.35	109.52	0.060
Total dietary fiber (g/1000 kcal)	6.70	2.23	6.23	1.93	**0.012**	6.82	2.23	6.06	1.91	**<0.001**	6.49	2.18	6.66	2.13	0.34

*Note*: Data are expressed as *n*, %, or mean, standard deviation. *p*‐values less than 0.05 are highlighted in bold.

Abbreviations: BMI, body mass index; %E, % of energy.

### 3.2 Obesity‐related anthropometric characteristics of the *ADRB3* genotype

The Trp64Trp genotype had an overall frequency of 64.5%, whereas the frequencies of Trp64Arg heterozygote and Arg64Arg homozygote were 31.8% and 3.7%, respectively (Table 2). The genotype frequency data were not different from those that were expected based on the Hardy‐Weinberg equilibrium (*p = *0.79). Moreover, there were no significant differences in BMI, WC, and BF percentages among the *ADRB3* genotypes.

**TABLE 2:** Obesity‐related anthropometric characteristics of the study participants by *ADRB3* genotype

	*ADRB3* genotype				*p*‐value	
	Trp64Trp	Trp64Arg	Arg64Arg	Trp64Arg + Arg64Arg	*p*1	*p*2
Number (%)	387 (64.5)	191 (31.8)	22 (3.7)	213 (35.5)		
BMI (kg/m^2^)	23.3 (3.2)	23.3 (3.2)	22.2 (3.4)	23.2 (3.2)	0.28	0.66
Waist circumference (cm)	83.9 (9.0)	84.1 (8.6)	82.6 (10.1)	84.0 (8.7)	0.75	0.91
Body fat (%)	29.8 (7.8)	29.0 (7.4)	29.5 (7.0)	29.1 (7.4)	0.49	0.24

*Note*: Data are expressed as *n* (%) or mean (standard deviation); *p*1, *p*‐value for ANOVA (Trp64Trp vs. Trp64Arg vs. Arg64Arg); *p*2, *p*‐value for Student *t* test (Trp64Trp vs. Arg64 carriers).

Abbreviations: *ADRB3*, β3‐adrenergic receptor; BMI, body mass index.

### 3.3 Association between overweight and related factors

Multivariable logistic regression analysis is presented in Table 3. The Trp64Arg polymorphism of ADRB3 was not significantly correlated with increased BMI, WC, or BF percentage; however, it showed a tendency to be associated with elevated BMI. Age was associated with increased BF percentage (odds ratio [OR] 1.035, 95% confidence interval [CI] 1.017–1.054), and females were observed to be associated with lower WC (OR: 0.255, 95% CI: 0.149–0.437). Among the lifestyle‐related factors, former smoking was significantly associated with WC (OR: 1.815, 95% CI: 1.054–3.125). Higher energy (OR: 1.001, 95% CI: 1.000–1.001) and sodium consumption (OR: 1.001, 95% CI: 1.000–1.001) were significantly associated with increased WC. By contrast, animal protein intake was inversely associated with WC (OR: 0.848, 95% CI: 0.733–0.980) and BF (OR: 0.837, 95% CI: 0.736–0.951).

**TABLE 3:** Factors associated with overweight based on a logistic regression analysis

	BMI			Waist circumference			Body fat		
	OR	95% CI	*p*‐value	OR	95% CI	*p*‐value	OR	95% CI	*p*‐value
Age (+1 year)	1.004	0.985–1.024	0.67	0.996	0.976–1.017	0.73	1.035	1.017–1.054	**<0.001**
Female	0.903	0.528–1.544	0.71	0.255	0.149–0.437	**<0.001**	1.185	0.721–1.948	0.50
Smoking status									
Former (vs. never)	1.567	0.910–2.699	0.11	1.815	1.054–3.125	**0.031**	1.431	0.862–2.376	0.17
Current (vs. never)	1.449	0.791–2.654	0.23	1.092	0.590–2.022	0.78	0.996	0.568–1.746	0.99
No regular exercise	1.122	0.766–1.645	0.55	1.466	0.986–2.178	0.059	1.073	0.757–1.522	0.69
Drinking habits	0.614	0.305–1.234	0.17	0.549	0.277–1.088	0.086	0.399	0.206–0.773	**0.006**
ADRB3 rs4994									
Trp64Trp (vs. Arg64Arg)	3.530	0.800–15.57	0.096	1.000	0.342–2.923	1.00	1.217	0.485–3.052	0.68
Trp64Arg (vs. Arg64Arg)	3.810	0.848–17.11	0.081	1.085	0.361–3.261	0.89	1.370	0.532–3.523	0.51
Dietary intake									
Total energy (+1 kcal/day)	1.000	1.000–1.001	0.057	1.001	1.000–1.001	**0.003**	1.000	1.000–1.000	0.49
Animal protein (+1%E)	0.880	0.765–1.011	0.071	0.848	0.733–0.980	**0.026**	0.837	0.736–0.951	**0.007**
Vegetable protein (+1%E)	1.014	0.750–1.372	0.93	0.908	0.661–1.246	0.55	0.918	0.701–1.202	0.53
Animal fat (+1%E)	1.040	0.948–1.140	0.41	1.009	0.915–1.113	0.86	1.028	0.945–1.118	0.52
Vegetable fat (+1%E)	0.990	0.928–1.057	0.77	0.995	0.930–1.063	0.87	0.996	0.939–1.057	0.90
Carbohydrate (+1%E)	0.982	0.939–1.027	0.42	0.980	0.937–1.025	0.37	0.952	0.913–0.993	**0.023**
Sodium (+1 mg/1000 kcal)	1.000	1.000–1.001	0.14	1.001	1.000–1.001	**0.003**	1.000	1.000–1.001	0.38
Calcium (+1 mg/1000 kcal)	1.001	0.998–1.004	0.57	1.001	0.998–1.004	0.55	1.002	0.999–1.004	0.20
Total dietary fiber (1 g/1000 kcal)	0.897	0.768–1.047	0.17	0.946	0.806–1.110	0.50	0.976	0.852–1.117	0.72

*Note*: *p*‐values less than 0.05 are highlighted in bold.

Abbreviations: ADRB3, β3‐adrenergic receptor; BMI, body mass index; CI, confidence interval; %E, % of energy; OR, odds ratio.

When a stepwise selection with backward elimination of predictors was further performed, the Trp64Arg genotype of ADRB3 tended to be associated with increased BMI; however, the association was not statistically significant (Table 4). Quitting smoking was associated with increased BMI (OR: 1.812, 95% CI: 1.185–2.772) and WC (OR: 1.726, 95% CI: 1.019–2.924). A lack of regular exercise or dietary intake of total energy and sodium were associated with increased WC. Conversely, daily animal protein intake displayed a negative association with WC (OR: 0.918, 95% CI: 0.854–0.987) and BF (OR: 0.899, 95% CI: 0.831–0.972).

**TABLE 4:** Factors associated with overweight based on a logistic regression analysis (stepwise selection with backward elimination)

BMI			
	OR	95% CI	*p*‐value
Smoking status			
Former (vs. never)	1.812	1.185–2.772	0.006
Current (vs. never)	1.689	1.061–2.688	0.027
ADRB3 rs4994			
Trp64Trp (vs. Arg64Arg)	3.794	0.867–16.605	0.077
Trp64Arg (vs. Arg64Arg)	4.201	0.945–18.682	0.059

Waist circumference			
	OR	95% CI	*p*‐value
Female (vs. Male)	0.251	0.151–0.418	<0.001
Smoking status			
Former (vs. never)	1.726	1.019–2.924	0.042
Current (vs. never)	1.046	0.594–1.842	0.875
No regular exercise	1.575	1.073–2.311	0.020
Dietary intake			
Total energy (+1 kcal/day)	1.000	1.000–1.001	0.010
Animal protein (+1%E)	0.918	0.854–0.987	0.021
Sodium (+ mg/100 kcal)	1.001	1.000–1.001	0.012

Body fat			
	OR	95% CI	*p*‐value
Age (+1 year)	1.038	1.021–1.055	<0.001
Drinking habits	0.399	0.240–0.663	<0.001
Dietary intake			
Animal protein (+1%E)	0.899	0.831–0.972	0.007
Carbohydrate (+1%E)	0.948	0.917–0.979	0.001

Abbreviations: ADRB3, β3‐adrenergic receptor; BMI, body mass index; CI, confidence interval; %E, % of energy; OR, odds ratio.

In the multivariable logistic regression analysis, the Trp64Arg polymorphism was not associated with any of the three obesity‐related indicators when treated with an additive model (Tables S1 and S2 in Supporting Information S1).

## 4 DISCUSSION

The present study demonstrated that the ADRB3 Trp64Arg polymorphism was not associated with increased BMI, WC, and BF percentages in community‐dwelling middle‐aged and older Japanese individuals. Instead, modifiable environmental factors were associated with obesity‐related parameters—physical inactivity, daily energy intake, and sodium were positively associated with WC, whereas animal protein intake was negatively associated with WC and BF percentage. Quitting the habit of smoking was also associated with increased BMI and WC. These results suggest that the main factors responsible for the increased body weight and fat mass in Japanese middle‐aged and older people are not *ADRB3* gene polymorphisms but the lifestyle that they follow.

Previous epidemiological studies have shown mixed results on the relationship between the *ADRB3* Trp64Arg polymorphism and obesity and its related parameters.^5^, ^6^, ^7^, ^8^, ^9^, ^10^, ^11^, ^12^, ^15^, ^16^, ^17^ A meta‐analysis report that examined study results on heterogeneous sources suggested that, on average, larger studies, studies in non‐selected populations, and studies in normal‐weight individuals tended to report smaller associations than studies conducted with smaller, selected, or obese populations.^11^ The present study population was selected from a comprehensive survey conducted in a single town and exhibited a relatively lower mean BMI (23 kg/m^2^). Consequently, these characteristics might have led to an insignificant relationship between the *ADRB3* polymorphism and obesity‐related parameters in the present study; nevertheless, this polymorphism demonstrated a tendency to be associated with BMI. Another possibility is that the *ADRB3* Trp64Arg polymorphism by itself might not have a clinically relevant function, but variations in this gene might be linked to an unidentified mutation. Indeed, certain in vitro studies have reported that the expression of *ADRB3* Trp64Arg polymorphism in CHO or HEK293 cells did not affect the efficacy or potency of cAMP accumulation of its agonists or the ability of these agonists to bind to their receptors.^33^, ^34^ Nonetheless, the functional significance and clinical importance of the *ADRB3* Trp64Arg polymorphism still remain to be clarified.

In the present study, the association of *ADRB3* gene polymorphisms and lifestyle‐related factors with obesity‐related parameters was examined in a multivariate model. Common risk factors, such as increased energy intake and physical inactivity were related to WC. These results were consistent with the primary findings of several management guidelines for obesity, including lifestyle changes, dietary modifications, and increased physical activity.^35^, ^36^, ^37^ Notably, cessation of smoking was also significantly associated with BMI and WC. Previously, several studies have indicated that the risk of obesity decreased in smokers; however, it was observed to increase after they quit smoking.^38^, ^39^, ^40^ Notably, a recent prospective cohort study has shown that although smoking cessation was accompanied with weight gain and an increased short‐term risk of type 2 diabetes, it did not mitigate the benefits in reducing cardiovascular and all‐cause mortality.^41^ While there is a possibility of reverse causality in the present study indicating that smoking cessation could have potentially occurred because the participants had encountered health problems, results in the present study suggest the importance of lifestyle modifications, including reducing energy intake and increasing physical activity in individuals who smoke and those who quit smoking, to control their weight appropriately.

In the present study, a daily intake of proteins, mainly animal protein among the energy‐yielding macronutrients, was negatively associated with WC and BF. This finding is consistent with the effect of proteins on energy homeostasis at the total protein level—proteins can participate in satiety and thermogenic processing involved in food intake and utilization.^3^ Proteins account for 20%–30% of the estimated diet‐induced thermogenesis and 99–153 kJ/ATP of ATP efficiency compared with 5%–10% and 91 kJ/ATP for glucose, and 0%–3% and 96 kJ/ATP for some fatty acids.^3^ Moreover, diets rich in proteins have been shown to reduce hunger perception and increased expenditure of β‐hydroxybutyrate levels, contributing to a decrease in appetite.^3^ At the protein quality level, inconsistent results have been reported concerning the relationship between proteins and WC or BF. For example, a recent prospective cohort study conducted in Australia has shown that an intake of animal protein was associated with increased WC and incident metabolic syndrome, while that of vegetable protein was not.^42^ A recent systematic review demonstrated that only a few observational studies associated animal protein intake with increased WC and body weight were performed in adults, and no differential effect was observed for vegetable proteins compared to animal proteins on body composition.^43^ The discrepancy in the animal protein‐related findings between the present study and previous studies, mainly from the western countries, might be attributable to the difference in the percentage of energy derived from animal proteins, which was higher in the western studies (median intake 11%–14%, expressed in energy percentage)^42^, ^44^ than in the present study (median intake 8.5%). The discrepancy might also be due to a difference in the main dietary source of animal protein, which was mainly red and processed meat in the western studies versus fish in the present study that was conducted at a rural coastal area in Japan. Furthermore, confounding factors, such as physical activity and energy intake, might not have been considered in the previous observational studies.^43^ Taken together, further longitudinal studies are required to provide insights into the effects of dietary protein intake and its quality on fat mass.

The present study has several limitations. First, the design was cross‐sectional, implying that a causal relationship could not be established between environmental factors, including nutrient intake and obesity‐related parameters. Second, only the *ADRB3* gene polymorphism was analyzed as a candidate gene for overweight and obesity in this relatively small genome cohort. However, several other obesity‐related candidate genes exist, such as leptin, leptin receptor, melanocortin 4 receptor,^1^, ^2^ uncoupling protein‐1,^45^ and fat mass and obesity‐associated protein (FTO)^46^ were not taken into account. Furthermore, investigations are required to elucidate the genetic factors associated with obesity using a large‐scale sample size. Third, the data on daily intake of energy, macronutrients, and minerals were obtained from the BDHQ, which comprises a limited number of food and beverage items and does not provide an accurate estimate of absolute dietary intake. Furthermore, these data may have been affected by the recall bias. Fourth, among the factors known to be associated with obesity,^1^ differences in socioeconomic status and certain measures of sleep and gut microbiota were not included the present study. Finally, there is a possibility of both a selection bias as well as a treatment effect bias, given that participants in the Shika study were volunteers and might have been more health‐conscious than the general population.

In conclusion, this cross‐sectional study reported that there was no statistically significant difference in all the overweight/obesity indicators between phenotypic groups of the Arg64 allele of the *ADRB3* gene. Instead, lifestyle‐related factors have a greater impact on increased body weight and adiposity in the general Japanese population aged ≥40 years. It appears that modifications in diet and lifestyle can overcome the effect of the *ADRB3* Trp64Arg gene polymorphism on overweight and obesity.

## CONFLICT OF INTEREST

The authors declare no conflicts of interest.

## AUTHOR CONTRIBUTIONS

Conceptualization, Akinori Hara and Hiroyuki Nakamura; methodology, Akinori Hara, Phat Minh Nguyen, and Hiroyuki Nakamura; formal analysis, Akinori Hara, Phat Minh Nguyen, Takehiro Sato, and Kazuyoshi Hosomichi; investigation, Akinori Hara, Phat Minh Nguyen, Takehiro Sato, and Kazuyoshi Hosomichi; resources, Atsushi Tajima and Hiroyuki Nakamura; data curation, Akinori Hara and Phat Minh Nguyen; writing—original draft preparation, Akinori Hara and Phat Minh Nguyen; writing—review and editing, Akinori Hara, Hiromasa Tsujiguchi, Oanh Kim Pham, Masaharu Nakamura, Yohei Yamada, Keita Suzuki, Fumihiko Suzuki, Takayuki Kannon, and Hiroyuki Nakamura, Yasuhiro Kambayashi, Yukari Shimizu, Thao Thi Thu Nguyen, Sakae Miyagi, Takehiro Sato, Takayuki Kannon, Kazuyoshi Hosomichi, Atsushi Tajima, and Hiroyuki Nakamura; supervision, Hiroyuki Nakamura; project administration, Hiromasa Tsujiguchi and Hiroyuki Nakamura; and funding acquisition, Akinori Hara and Hiroyuki Nakamura; All authors have read and agreed to the published version of the manuscript.

## Supporting information

Supplementary Material
