# Supplementary material for: Effect of β3‐adrenergic receptor gene polymorphism and lifestyle on overweight Japanese rural residents: A cross‐sectional study
Source: Obes Sci Pract. 2021 Sep 21;8(2):199–207. doi: 10.1002/osp4.560 (PMC8976547; doi:10.1002/osp4.560)
Supplement: Supplementary file 1 — Supplementary Material [file OSP4-8-199-s001.docx]

**Supplementary Table 1. Factors associated with overweight based on a logistic regression analysis**

|  | BMI | | |  | Waist circumference | | |  | Body fat | | |
| --- | --- | --- | --- | --- | --- | --- | --- | --- | --- | --- | --- |
|  | OR | 95% CI | p value |  | OR | 95% CI | p value |  | OR | 95% CI | p value |
| Age (+1 year) | 1.005 | 0.986 - 1.025 | 0.62 |  | 0.996 | 0.977 - 1.017 | 0.73 |  | 1.035 | 1.017 - 1.054 | <0.001 |
| Female | 0.879 | 0.514 - 1.503 | 0.64 |  | 0.254 | 0.149 - 0.435 | <0.001 |  | 1.171 | 0.713 - 1.923 | 0.53 |
| Smoking status |  |  |  |  |  |  |  |  |  |  |  |
| Former (vs. never) | 1.574 | 0.913 - 2.714 | 0.10 |  | 1.817 | 1.055 - 3.127 | 0.031 |  | 1.433 | 0.864 - 2.379 | 0.16 |
| Current (vs. never) | 1.442 | 0.788 - 2.639 | 0.24 |  | 1.091 | 0.590 - 2.020 | 0.78 |  | 0.993 | 0.567 - 1.739 | 0.98 |
| No regular exercise | 1.124 | 0.767 - 1.646 | 0.55 |  | 1.466 | 0.986 - 2.178 | 0.059 |  | 1.076 | 0.759 - 1.525 | 0.68 |
| Drinking habits | 0.593 | 0.296 - 1.186 | 0.14 |  | 0.545 | 0.275 - 1.08 | 0.082 |  | 0.394 | 0.203 - 0.761 | 0.006 |
| Trp64Arg polymorphism of ADRB3 | 0.886 | 0.632 - 1.243 | 0.48 |  | 1.053 | 0.746 - 1.487 | 0.77 |  | 1.037 | 0.768 - 1.401 | 0.81 |
| Dietary intake |  |  |  |  |  |  |  |  |  |  |  |
| Total energy (+1 kcal/day) | 1.000 | 1.000 - 1.001 | 0.048 |  | 1.001 | 1.000 - 1.001 | 0.003 |  | 1.000 | 1.000 - 1.000 | 0.47 |
| Animal protein (+1%E) | 0.879 | 0.765 - 1.011 | 0.070 |  | 0.848 | 0.733 - 0.98 | 0.026 |  | 0.837 | 0.736 - 0.951 | 0.007 |
| Vegitable protein (+1%E) | 1.015 | 0.749 - 1.374 | 0.93 |  | 0.907 | 0.661 - 1.246 | 0.55 |  | 0.918 | 0.701 - 1.202 | 0.53 |
| Animal fat (+1%E) | 1.035 | 0.944 - 1.135 | 0.46 |  | 1.008 | 0.914 - 1.112 | 0.87 |  | 1.026 | 0.944 - 1.116 | 0.54 |
| Vegitable fat (+1%E) | 0.992 | 0.930 - 1.058 | 0.81 |  | 0.995 | 0.931 - 1.064 | 0.88 |  | 0.996 | 0.939 - 1.057 | 0.91 |
| Carbohydrate (+1%E) | 0.981 | 0.938 - 1.025 | 0.39 |  | 0.98 | 0.937 - 1.025 | 0.37 |  | 0.952 | 0.913 - 0.993 | 0.022 |
| Sodium (+1 mg/1000 kcal) | 1.000 | 1.000 - 1.001 | 0.13 |  | 1.001 | 1.000 - 1.001 | 0.003 |  | 1.000 | 1.000 - 1.001 | 0.38 |
| Calcium (+1 mg/1000 kcal) | 1.001 | 0.998 - 1.004 | 0.50 |  | 1.001 | 0.998 - 1.004 | 0.54 |  | 1.002 | 0.999 - 1.004 | 0.18 |
| Total dietary fiber (1 g/1000 kcal) | 0.893 | 0.765 - 1.043 | 0.15 |  | 0.945 | 0.805 - 1.109 | 0.49 |  | 0.974 | 0.851 - 1.115 | 0.71 |

Trp64Arg polymorphism was used as an ordinal variable; 0, Trp64Trp; 1, Trp64Arg, and 2, Arg64Arg.

BMI, body mass index; OR, odds ratio; CI, confidence interval; ADRB3, β3-adrenergic receptor; %E, % of energy

**Supplementary Table 2.** **Factors associated with overweight based on a logistic regression analysis (stepwise selection with backward elimination)**

| BMI | | | |  | Waist circumference | | | |  | Body fat | | | |
| --- | --- | --- | --- | --- | --- | --- | --- | --- | --- | --- | --- | --- | --- |
|  | OR | 95% CI | p value |  |  | OR | 95% CI | p value |  |  | OR | 95% CI | p value |
| Smoking status |  |  |  |  | Female (vs. Male) | 0.251 | 0.151 - 0.418 | <0.001 |  | Age (+1 years) | 1.038 | 1.021 - 1.055 | <0.001 |
| Former (vs. never) | 1.837 | 1.203 - 2.804 | 0.005 |  | Smoking status |  |  |  |  | Drinking habits | 0.399 | 0.240 - 0.663 | <0.001 |
| Current (vs. never) | 1.665 | 1.048 - 2.644 | 0.031 |  | Former (vs. never) | 1.726 | 1.019 - 2.924 | 0.042 |  | Dietary intake |  |  |  |
|  |  |  |  |  | Current (vs. never) | 1.046 | 0.594 - 1.842 | 0.88 |  | Animal protein (+1%E) | 0.899 | 0.831 - 0.972 | 0.007 |
|  |  |  |  |  | No regular exercise | 1.575 | 1.073 - 2.311 | 0.020 |  | Carbohydrate (+1%E) | 0.948 | 0.917 - 0.979 | 0.001 |
|  |  |  |  |  | Dietary intake |  |  |  |  |  |  |  |  |
|  |  |  |  |  | Total energy (+1 kcal/day) | 1.000 | 1.000 - 1.001 | 0.010 |  |  |  |  |  |
|  |  |  |  |  | Animal protein (+1%E) | 0.918 | 0.854 - 0.987 | 0.021 |  |  |  |  |  |
|  |  |  |  |  | Sodium (+1 mg/1000 kcal) | 1.001 | 1.000 - 1.001 | 0.012 |  |  |  |  |  |

BMI, body mass index; OR, odds ratio; CI, confidence interval; %E, % of energy
